# Analysis of rational use and misuse of antibiotics in emergency departments: A cross-sectional study

**DOI:** 10.1371/journal.pone.0350138

**Published:** 2026-06-03

**Authors:** Huseyin Gurbuz, Selda Aslan, Muhammet Emin Doğan, Mehmet Ali Çevik

**Affiliations:** 1 Department of Emergency Medicine, Harran University Faculty of Medicine, Şanlıurfa, Türkiye; 2 Department of Infectious Diseases and Clinical Microbiology, Gaziantep Islam Science and Technology University, Gaziantep, Türkiye; 3 Emergency Department, 25 Aralık State Hospital, Gaziantep, Türkiye; 4 Department of Emergency Medicine, Gaziantep University Faculty of Medicine, Gaziantep, Türkiye; UCSI University, MALAYSIA

## Abstract

**Objective:**

To assess antibiotic prescribing frequency and the appropriateness of prescriptions based on established rational antibiotic use principles among adult patients evaluated in the emergency department.

**Methods:**

This retrospective, cross-sectional study was conducted in the emergency department of a tertiary university hospital between 1 January and 30 June 2022. Adult patients (≥18 years) who received or were prescribed antibiotics were included. Demographic, clinical, and microbiological data were retrieved from the hospital information management system. Prescriptions were evaluated against rational antibiotic use criteria defined by the World Health Organization (WHO) and the Turkish Medicines and Medical Devices Agency (TİTCK).

**Results:**

A total of 595 patients were analyzed (62.4% female; mean age, 31.1 ± 14.2 years). Antibiotics were prescribed in 95.8% of encounters. Amoxicillin–clavulanic acid was the most commonly used agent (58.7%), followed by amoxicillin (8.7%) and metronidazole (5.6%). Microbiological culture sampling was conducted in only 0.8% of cases. Among patients who received antibiotic therapy (n = 570), 87.2% of prescriptions were classified as appropriate and 12.8% were classified as inappropriate according to guideline-based evaluation. In the overall cohort, 4.2% of patients did not receive antibiotic therapy. Most treated patients received a single antibiotic (95.8%), whereas dual antibiotic therapy was infrequently used (4.0%).

**Conclusion:**

Antibiotic prescribing in the emergency department was notably high, accompanied by critically low rates of microbiological investigation. The findings demonstrate a persistent reliance on empirical therapy. Enhancing diagnostic support, promoting culture-guided treatment, and incorporating routine antimicrobial de-escalation strategies may help strengthen rational antibiotic use and warrant further evaluation.

## 1. Introduction

Rational drug use (RDU) refers to the practice of providing patients with medications that are appropriate to their clinical needs and individual characteristics, administered in correct doses and for an adequate duration, while minimizing costs for both the individual and society [1]. Failure to adhere to these principles results in significant individual and public health consequences, including reduced treatment adherence, adverse drug interactions, increased antimicrobial resistance, and escalating healthcare expenditures [2].

Emergency departments (EDs) represent clinical settings where inappropriate medication and antibiotic use is particularly common, largely due to high patient volumes, time constraints, diagnostic uncertainty, and the frequent need for empirical treatment decisions [3]. Previous studies have shown that approximately 15% of patients presenting to EDs receive antimicrobial prescriptions, many of which are issued without clear clinical indications. This practice substantially contributes to the emergence and spread of antimicrobial resistance, which has become a major global public health concern [4].

Rational antibiotic use is therefore critical not only for optimizing individual patient outcomes but also for preserving the effectiveness of existing antimicrobial agents at the population level. Inappropriate antimicrobial prescribing accelerates the development of resistant microorganisms, increases morbidity and mortality, and imposes a growing economic burden on healthcare systems worldwide [5,6]. In Türkiye, multiple studies have reported that awareness and implementation of rational drug use principles among both healthcare professionals and the general population remain suboptimal [2].

Assessing antibiotic prescribing practices in emergency departments is particularly important, as empirical antibiotic initiation is frequently preferred in this setting. Evaluations should focus on the appropriateness of clinical indications, antibiotic selection, dosing regimens, treatment duration, and opportunities for de-escalation, in accordance with evidence-based guidelines [7,8]. Such assessments are essential for strengthening national rational drug use policies and antimicrobial stewardship strategies.

The objective of this study was to retrospectively evaluate the appropriateness of antibiotic prescriptions in patients presenting to the emergency department based on rational antibiotic use principles and to identify factors associated with inappropriate prescribing. The findings are intended to contribute to improving antibiotic prescribing practices in emergency care, supporting efforts to combat antimicrobial resistance, and promoting more efficient use of healthcare resources.

This study can also be interpreted as a drug utilization review of antibiotic prescribing patterns in the emergency department, aligned with antimicrobial stewardship principles and World Health Organization recommendations for rational antibiotic use.

## 2. Methods

### 2.1. Study design and setting

This retrospective, cross-sectional observational study was conducted in the emergency department of a tertiary-care university hospital. Ethical approval was obtained from a local institutional clinical research ethics committee (Approval No: 2022/312, Date: September 27, 2022). The study period covered a six-month interval between January 1, 2022, and June 30, 2022. This study should be interpreted as a retrospective observational chart review evaluating associations between clinical variables and inappropriate antibiotic prescribing rather than establishing causal relationships.

The study population consisted of adult patients aged 18 years and older who were prescribed antibiotics or received antibiotic therapy during their emergency department visit. Patients who did not receive antibiotics, were under 18 years of age, or had incomplete medical records were excluded. A total of 595 patients meeting the inclusion criteria were evaluated.

### 2.2. Data collection process

Data were retrospectively obtained from the Hospital Information Management System (HIMS). Emergency department admission forms, physician notes, laboratory results, antibiotic prescriptions, and discharge summaries were reviewed.

Study data were accessed for research purposes between 15/01/2023 and 20/01/2023.

A standardized electronic data collection form developed by the researchers was used. The form was piloted on 20 randomly selected patient records prior to the study, and necessary revisions were made. Data extraction was performed independently by two researchers experienced in emergency medicine, and any discrepancies were reassessed by a third expert. Appropriateness assessments were independently performed by two investigators, and disagreements were resolved by consensus with a third senior reviewer. Incomplete records lacking key variables required for analysis were excluded, and duplicate entries were identified and removed during the data cleaning process prior to statistical analysis.

### 2.3. Defined variables

The variables analyzed in the study were categorized as follows:

- **Demographic variables:** age, sex
- **Clinical variables:** chief complaint, diagnosis, vital signs, laboratory values
- **Antibiotic-related variables:** type of antibiotic, dose, route of administration, duration of therapy, clinical indication, microbiological culture availability and results, and whether de-escalation was performed

Antibiotic appropriateness was evaluated based on clinical indication, antimicrobial spectrum, dose, treatment duration, and incorporation of available microbiological data into treatment decisions, in accordance with World Health Organization and national antibiotic use guidelines.

Failure to obtain microbiological cultures was not automatically classified as inappropriate prescribing but was considered a diagnostic stewardship deficiency when cultures were clinically indicated. Appropriateness of antibiotic prescriptions was determined using predefined criteria derived from WHO rational drug use principles and national antimicrobial guidelines, including clinical indication, recommended first-line agents, antimicrobial spectrum, dosing regimen, and treatment duration. Inappropriate antibiotic use was defined as prescribing antibiotics without a medical indication, incorrect dose or duration, or unnecessary selection of broad-spectrum agents.

### 2.4. Ethical statement

The study was conducted in accordance with the ethical principles of the Declaration of Helsinki (2013). Ethical approval was obtained from an institutional Clinical Research Ethics Committee (Approval No: 2022/312, Date: September 27, 2022). Written institutional permission was also secured from the hospital administration where the study was conducted.

Due to the retrospective design of the study, the requirement for informed consent was waived by the ethics committee. All data were anonymized prior to analysis and used solely for scientific purposes.

### 2.5. Statistical analysis

- Data analysis was performed using IBM SPSS Statistics for Windows, Version 25.0 (IBM Corp., Armonk, NY, USA).
- Categorical variables were presented as frequencies (n) and percentages (%). Continuous variables were expressed as mean ± standard deviation (SD) or median (minimum–maximum). Pearson’s chi-square test or Fisher’s exact test was used for categorical variables, while Student’s t-test or Mann–Whitney U test was applied for continuous variables, as appropriate.
- Univariate logistic regression analysis was performed to identify factors associated with inappropriate antibiotic use. Variables with a p-value <0.10 in univariate analysis were included in the multivariate logistic regression model to avoid excluding potentially relevant confounders. The Forward Likelihood Ratio method was used, and odds ratios (ORs) with 95% confidence intervals (CIs) were calculated.
- Statistical significance was defined as p < 0.05.
- Clinically relevant variables identified from prior literature and clinical reasoning were also considered in the multivariable model to minimize potential confounding.

## 3. Results

A total of 595 adult patients were included in the study. The mean age was 31.1 ± 14.2 years (range: 18–84 years). Of the participants, 62.4% were female (n = 371) and 37.6% were male (n = 224). Emergency department visits occurred during working hours in 59.8% of cases and outside working hours in 40.2%. Most patients were triaged to the green area (98.8%), while 1.2% were classified as red area cases (Table 1).

**Table 1:** Demographic characteristics, diagnoses, and infection focus of the study population (n = 595).

Variable	Minimum-maximum	Median	Mean ± SD or n (%)
Age (years)	18.0-84.0	25.0	31.1 ± 14.2
**Sex**			
Male	–	–	224 (37.6%)
Female	–	–	371 (62.4%)
**Time of ED presentation**			
Outside working hours	–	–	239 (40.2%)
Working hours	–	–	356 (59.8%)
**Triage category**			
Red area	–	–	7 (1.2%)
Green area	–	–	588 (98.8%)
**Diagnoses**			
Upper urinary tract infection	–	–	3 (0.5%)
Lower urinary tract infection	–	–	36 (6.1%)
Diarrhea	–	–	40 (6.7%)
Abscess	–	–	3 (0.5%)
Skin and soft tissue infection	–	–	22 (3.7%)
Bone and joint infection	–	–	1 (0.2%)
Eye infection	–	–	3 (0.5%)
Other infections	–	–	4 (0.7%)
**Infection focus**			
None identified	–	–	308 (51.8%)
Gastrointestinal system	–	–	45 (7.6%)
Respiratory system	–	–	166 (27.9%)
ENT	–	–	44 (7.4%)
Skin	–	–	8 (1.3%)
Urinary tract	–	–	22 (3.7%)
Orthopedic	–	–	1 (0.2%)
Unknown	–	–	1 (0.2%)
CRP (mg/L)	0.2-177.9	5.0	19.5 ± 36.9
WBC (×10³/µL)	1.4-25.5	9.0	9.5 ± 3.7
Neutrophils (%)	0.7-95.4	8.9	30.8 ± 30.5
Lymphocytes (%)	0.2-53.4	3.3	11.3 ± 13.0
Urine culture obtained	–	–	5 (0.8%)
Wound prophylaxis	–	–	5 (0.8%)

**Abbreviations:** CRP, C-reactive protein; ED, emergency department; ENT, ear-nose-throat; SD, standard deviation; WBC, white blood cell count.

Regarding diagnostic distribution, the most frequently identified infections were diarrhea (6.7%), lower urinary tract infections (6.1%), and skin and soft tissue infections (3.7%). No identifiable source of infection was detected in 51.8% of patients. Among cases with a defined focus, respiratory system involvement (27.9%) and gastrointestinal system involvement (7.6%) were the most common (Table 1).

All patients were managed on an outpatient basis. Antibiotics were prescribed to 95.8% of patients (n = 570), whereas 4.2% (n = 25) did not receive antibiotic therapy. Subsequent analyses of antibiotic type, number of agents, route of administration, and treatment duration were restricted to patients who received antibiotic therapy (n = 570). Among patients receiving antibiotics, 95.8% (n = 546) were prescribed a single antibiotic, while 4.0% (n = 23) received dual antibiotic therapy. The median time from emergency department admission to antibiotic prescription was 22 minutes, with a mean of 52.9 ± 93.4 minutes (Table 2).

**Table 2:** Antibiotic prescribing process and treatment characteristics among patients who received antibiotics (n = 570).

Variable		n (%)
**Number of antibiotics prescribed**		
	Single antibiotic	546 (95.8)
	Two antibiotics	23 (4.0)
	Three or more antibiotics	1 (0.2)
**Route of administration**		
	Oral	488 (85.6)
	Intravenous	82 (14.4)
**Duration of prescribed therapy**		
	≤5 days	421 (73.9)
	6–10 days	149 (26.1)

Abbreviation: n, number of patients.

The most frequently used dosing regimen was twice daily (2 × 1), observed in 97.3% of cases. Once-daily (1 × 1) dosing was used in 2.5%, and a four-times-daily regimen (2 × 2) was used in 0.2% of patients (Table 2).

Comparison between patients with appropriate and inappropriate antibiotic use revealed no significant differences in mean age, sex distribution, or triage category (p > 0.05). Presentation to the emergency department outside working hours was significantly more frequent in the inappropriate antibiotic use group (p = 0.039). Additionally, a diagnosis of lower urinary tract infection was significantly more common among patients with inappropriate antibiotic use (p = 0.001) (Table 3).

**Table 3:** Comparative analysis according to antibiotic appropriateness.

Variable	Antibiotic inappropriate (n = 78), Mean ± SD or n (%)	Median	Antibiotic appropriate (n = 517), Mean ± SD or n (%)	Median	p-value	Test
Age (years)	31.6 ± 14.9	23.0	31.0 ± 14.1	25.0	0.750	Mann-Whitney U
**Sex**					0.927	Chi-square
Male	29 (37.2%)	–	195 (37.7%)	–		
Female	49 (62.8%)	–	322 (62.3%)	–		
**Time of ED presentation**					0.039	Chi-square
Outside working hours	23 (29.5%)	–	216 (41.8%)	–		
Working hours	55 (70.5%)	–	301 (58.2%)	–		
**Triage category**					0.603	Chi-square/Fisher
Red area	0 (0.0%)	–	7 (1.4%)	–		
Green area	78 (100%)	–	510 (98.6%)	–		
Any infection present	41 (52.6%)	–	246 (48.0%)	–	0.412	Chi-square
Upper urinary tract infection	0 (0.0%)	–	3 (0.6%)	–	1.000	Chi-square/Fisher
Lower urinary tract infection	11 (14.1%)	–	25 (4.8%)	–	0.001	Chi-square
Diarrhea	4 (5.1%)	–	36 (7.0%)	–	0.546	Chi-square
Abscess	0 (0.0%)	–	3 (0.6%)	–	1.000	Chi-square/Fisher
Skin and soft tissue infection	1 (1.3%)	–	21 (4.1%)	–	0.225	Chi-square
Bone and joint infection	0 (0.0%)	–	1 (0.2%)	–	1.000	Chi-square/Fisher
Eye infection	1 (1.3%)	–	2 (0.4%)	–	0.344	Chi-square
Other infections	2 (2.6%)	–	2 (0.4%)	–	0.085	Chi-square
CRP (mg/L)	13.8 ± 32.3	3.9	20.4 ± 37.5	5.1	0.053	Mann-Whitney U
WBC (×10³/µL)	8.7 ± 3.1	8.6	9.6 ± 3.8	9.0	0.148	Mann-Whitney U
Neutrophils (%)	32.0 ± 31.1	8.9	30.7 ± 30.4	9.0	0.888	Mann-Whitney U
Lymphocytes (%)	11.8 ± 13.3	3.4	11.2 ± 13.0	3.2	0.847	Mann-Whitney U
Urine culture obtained	2 (2.6%)	–	3 (0.6%)	–	0.130	Chi-square
Wound prophylaxis	1 (1.3%)	–	4 (0.8%)	–	0.506	Chi-square
Time to antibiotic prescription (min)	63.3 ± 84.8	21.0	51.3 ± 94.7	22.0	0.407	Mann-Whitney U
**Number of antibiotics**					0.788	Chi-square
One	74 (94.9%)	–	494 (95.6%)	–		
Two	4 (5.1%)	–	22 (4.3%)	–		
≥ Three	0 (0.0%)	–	1 (0.2%)	–		
**Dosage regimen**					0.431	Chi-square
1 × 1	3 (3.8%)	–	12 (2.3%)	–		
2 × 1	75 (96.2%)	–	504 (97.5%)	–		
2 × 2	0 (0.0%)	–	1 (0.2%)	–		

**Abbreviations:** CRP, C-reactive protein; ED, emergency department; SD, standard deviation; WBC, white blood cell count. Fisher’s exact test was used when appropriate.

Multivariate logistic regression analysis identified presentation outside working hours and a diagnosis of lower urinary tract infection as independent factors associated with inappropriate antibiotic use (Table 3).

Microbiological evaluation showed that culture samples were obtained from only 0.8% of patients (n = 5), all of which were urine cultures. No microbiological sampling was performed in 99.2% of patients (n = 590) (Table 4).

**Table 4:** Microbiological sampling characteristics among the study population (n = 595).

Characteristic	Category	n (%)
**Microbiological culture sampling**	**Culture obtained**	5 (0.8)
	**No culture obtained**	590 (99.2)
	**Total**	595 (100.0)

Abbreviation: n, number of patients.

According to rational antibiotic use criteria, 83.5% of antibiotic prescriptions (n = 497) were classified as appropriate, while 12.3% (n = 73) were considered inappropriate (Table 5).

**Table 5:** Antibiotic appropriateness and distribution of antibiotic types among the study population (n = 595).

Characteristic	Category	n (%)
**Antibiotic appropriateness**	Appropriate	497 (83.5)
	Inappropriate	73 (12.3)
	No antibiotic therapy	25 (4.2)
**Types of antibiotics used**	Amoxicillin–clavulanate	349 (58.7)
	Amoxicillin	52 (8.7)
	Metronidazole	34 (5.7)
	Macrolides	27 (4.5)
	Third-generation cephalosporins	28 (4.7)
	Fluoroquinolones	26 (4.4)
	Other antibiotics	79 (13.3)
	**Total**	**595 (100.0)**

Abbreviation: n, number of patients.

Analyses of antibiotic appropriateness were restricted to patients who received antibiotic therapy (n = 570), whereas the no antibiotic therapy category was retained only in the overall cohort summary. Analysis of antibiotic classes demonstrated that amoxicillin–clavulanic acid was the most frequently prescribed agent (58.7%), followed by amoxicillin (8.7%), metronidazole (5.7%), third-generation cephalosporins (4.7%), macrolides (4.5%), and fluoroquinolones (4.4%). Other antibiotics accounted for 13.3% of prescriptions (Table 5).

Regarding laboratory findings, the median C-reactive protein level was 5,0 mg/L, with a mean value of 19.19 ± 36,42 mg/L. The median white blood cell count was 9.0 × 10³/µL, with a mean of 9.51 ± 3.71 × 10³/µL. Mean ± SD values for neutrophil and lymphocyte percentages were 31.44 ± 30.61% and 11.50 ± 13.07%, respectively. Detailed minimum and maximum values are presented in Table 6.

**Table 6:** Descriptive statistics of laboratory parameters.

Parameter	Minimum	Maximum	Mean ± SD
**CRP (mg/L)**	0.19	177.90	19.19 ± 36.42
**WBC (×10³/µL)**	1.37	25.45	9.51 ± 3.71
**Neutrophils (%)**	0.68	95.40	31.44 ± 30.61
**Lymphocytes (%)**	0.21	53.40	11.50 ± 13.07

**Abbreviations:** CRP, C-reactive protein; SD, standard deviation; WBC, white blood cell count.

## 4. Discussion

This study evaluated real-world antibiotic prescribing behavior among adult patients presenting to a tertiary emergency department and demonstrated a substantial reliance on empirical antimicrobial therapy, limited diagnostic testing, and moderate rates of inappropriate prescribing. Although 83.5% of prescriptions were formally guideline-concordant, the coexistence of inappropriate use and an extremely low microbiological culture sampling rate (0.8%) indicates that diagnostic-driven prescribing remains insufficiently integrated into emergency department (ED) workflow.

Previous studies have emphasized the central role of antimicrobial stewardship interventions in emergency departments and highlighted persistent challenges in translating stewardship principles into routine clinical practice [9–11]. In the present study, despite a high proportion of formally appropriate prescriptions, the near-absence of microbiological culture utilization suggests that similar stewardship challenges persist in this emergency care setting. Consistent with these observations, our findings indicate that high apparent prescribing appropriateness can coexist with substantial gaps in diagnostic stewardship. Zhang et al. reported antibiotic use in 89% of adult ED encounters in China, underscoring the global tendency toward empirical treatment in acute care environments [12]. Similarly, Lim et al. demonstrated that antibiotic prescribing decisions in emergency departments are strongly influenced by diagnostic uncertainty and time pressure, even in clinically uncomplicated presentations [13]. In our cohort, microbiological samples were obtained in only 0.8% of patients (n = 5), indicating that antibiotic decision-making was overwhelmingly empirical. This extremely low culture acquisition rate highlights a significant diagnostic stewardship gap and suggests that antibiotic prescribing decisions were largely empirical in this emergency department setting.

The contribution of diagnostic testing to antibiotic decision-making remains variable across emergency care systems. Studies evaluating rapid viral testing in emergency settings have suggested potential reductions in unnecessary antibiotic use; however, implementation remains inconsistent [14]. O’Connell et al. further demonstrated that point-of-care diagnostic strategies can influence prescribing behavior, though their integration into routine ED workflows remains limited [15]. The findings of the present study align with this literature, revealing minimal diagnostic support for antibiotic initiation in routine emergency practice.

Microbiological culture utilization emerged as the most pronounced stewardship deficiency. Only 0.8% of patients underwent culture sampling, a rate far below those reported in emergency department interventions specifically targeting diagnostic stewardship. Theophanous et al. reported culture acquisition rates exceeding 30% following implementation of a blood culture algorithm, accompanied by measurable improvements in antimicrobial use [16]. Similarly, Losier et al. demonstrated that structured antimicrobial stewardship protocols were associated with improved prescribing behavior and greater diagnostic engagement [17]. Bishop previously emphasized that inadequate microbiological testing in emergency departments promotes unnecessary broad-spectrum antibiotic use and accelerates antimicrobial resistance, a concern directly supported by our findings [18].

Several operational factors may underlie the low culture acquisition rate observed in this cohort, including high patient turnover, perceived low illness severity, and workflow prioritization favoring rapid disposition over diagnostic clarification. These practices contrast with recommendations outlined in the Sepsis-3 definitions and the Surviving Sepsis Campaign guidelines, which advocate obtaining appropriate cultures prior to antimicrobial initiation whenever clinically feasible [19,20]. The near absence of culture sampling observed in this study suggests that these evidence-based recommendations have not yet been robustly adopted in routine ED practice.

Amoxicillin–clavulanic acid was the most frequently prescribed antibiotic (58.7%), reflecting a widespread perception of broad-spectrum agents as “safe default” options in emergency settings. This prescribing pattern parallels findings from surveys indicating that awareness of antimicrobial resistance does not consistently translate into rational prescribing under conditions of time pressure and perceived patient demand [21]. Reliance on broad-spectrum therapy, even in uncomplicated infections, contributes to unnecessary antimicrobial exposure and resistance development. Antibiotic prescribing decisions in emergency departments are frequently influenced by diagnostic uncertainty and time pressure.

The implications of these findings extend beyond individual emergency departments. In the present study, the combination of a very high rate of empirical antibiotic prescribing and an extremely low frequency of microbiological culture acquisition highlights that inappropriate antibiotic use is driven not only by individual prescribing decisions but also by system-level and diagnostic limitations. Inappropriate antibiotic use has been associated with preventable adverse events, increased healthcare utilization, and the accelerating burden of antimicrobial resistance [22]. Global surveillance data further indicate that, if current prescribing patterns persist, antimicrobial resistance will continue to rise worldwide and constitute a major public health threat [23]. Evidence from diverse clinical settings demonstrates that antimicrobial stewardship interventions can significantly improve prescribing appropriateness, supporting the feasibility of system-level change.

In this context, strengthening antimicrobial stewardship practices in emergency departments emerges as an important area for further evaluation. Given the diagnostic stewardship gaps identified in our study, particularly the near-absence of culture-based confirmation, interventions focusing on diagnostic integration and structured reassessment may be especially relevant in similar high-volume emergency care environments. Previous studies have shown that structured stewardship programs, interdisciplinary collaboration, and protocolized reassessment can improve antibiotic prescribing quality in emergency settings [23]. Future prospective studies are warranted to evaluate the effectiveness of integrating diagnostic stewardship and structured de-escalation strategies into routine emergency department practice. These findings also emphasize the importance of strengthening antimicrobial stewardship strategies and aligning prescribing practices with World Health Organization recommendations for rational antibiotic use.

In summary, these findings underscore the need for systematic stewardship strategies focused on increasing microbiological testing, implementing rapid diagnostics, curbing default use of broad-spectrum agents, and ensuring structured reassessment and de-escalation. In this context, the observed prescribing patterns may contribute to ongoing antimicrobial stewardship challenges in both institutional and broader healthcare settings. These findings primarily reflect real-world antibiotic prescribing patterns observed in this emergency department setting and should be interpreted cautiously within the limitations of a single-center retrospective observational study, rather than as evidence of causal relationships or the effectiveness of antimicrobial stewardship interventions. They are also consistent with World Health Organization recommendations emphasizing rational antibiotic use, strengthened diagnostic stewardship, and culture-guided treatment strategies.

## 5. Conclusion

This study demonstrated that antibiotic use in emergency departments is widespread, while culture sampling rates are extremely low. Strengthening culture-based diagnostic approaches, standardizing specimen collection prior to antibiotic initiation, and implementing regular antimicrobial stewardship audits may help improve rational antibiotic use and warrant further evaluation. Our findings indicate that sustainable success requires not only individual awareness but also system-level improvements. These findings also emphasize the importance of strengthening antimicrobial stewardship strategies and aligning prescribing practices with World Health Organization recommendations for rational antibiotic use. These findings primarily reflect antibiotic prescribing patterns observed in this emergency department setting and should be interpreted cautiously within the limitations of a single-center retrospective observational study. These findings should be interpreted in the context of low-acuity ambulatory adult emergency department patients and should not be generalized to critically ill or hospitalized populations without caution.

## 6. Limitations

Although the single-center and retrospective design of the study limits its generalizability, the large sample size and the use of real-world emergency department data provide valuable insights into antibiotic prescribing practices. In addition, because the study population consisted almost entirely of low-acuity ambulatory adult patients, the findings may not reflect antibiotic prescribing patterns in critically ill or hospitalized emergency department populations. Direct access to the clinical rationale underlying physicians’ antibiotic prescribing decisions was not possible. Additionally, the absence of follow-up data limited the evaluation of the clinical outcomes associated with prescribed antibiotics. Because microbiological sampling was extremely limited in this cohort, the present study could not evaluate microbiology-guided therapy or antimicrobial de-escalation practices. Moreover, the lack of rapid diagnostic testing for viral and bacterial differentiation may have contributed to empirical antibiotic prescribing.

## Supporting information

S1 Dataset De-identified dataset used for statistical analysis of antibiotic prescribing patterns and rational antibiotic use in the emergency department. (XLSX)
